# Internal carotid artery dissection in a patient with Parkinson's disease after COVID-19 infection

**DOI:** 10.1016/j.ensci.2024.100529

**Published:** 2024-09-27

**Authors:** Takanobu Okubo, Hidehiro Ishikawa, Keita Matsuura, Asako Tamura, Koichi Miyashita, Maki Umino, Masayuki Maeda, Akihiro Shindo

**Affiliations:** a Department of Neurology, Mie University Graduate School of Medicine, Mie, Japan; b Deptartment of Radiology, Mie University Graduate School of Medicine, Mie, Japan; c Deptartment of Neuroradiology, Mie University Graduate School of Medicine, Mie, Japan

**Keywords:** Internal carotid artery dissection, COVID-19, Parkinson's disease, Dyskinesia, Stroke, Case report

## Abstract

**Background:**

Internal carotid artery (ICA) dissection is a relatively rare cause of acute ischemic stroke. Stretching and compression of ICA due to sudden acceleration, deceleration, and rotational forces are risk factors for ICA dissection. Severe acute respiratory syndrome coronavirus 2 (SARS-CoV-2) is believed to trigger an inflammatory response that exacerbates endothelial dysfunction and leads to arterial dissection. Although levodopa-induced cervical dyskinesia in Parkinson's disease often manifests as choreiform movement, dissection has not been reported in such patients.

**Case presentation:**

A 51-year-old man with Parkinson's disease (PD) presented with gradually worsening neck pain and transient aphasia 1 week after mild coronavirus disease 2019 (COVID-19) infection. The patient already had neck pain due to cervical spondylosis and presented with levodopa-induced cervical dyskinesia. Magnetic resonance imaging revealed acute ischemic stroke in the left parietal lobe and an intramural hematoma with an area of stenosis in the left ICA. The patient was diagnosed with left ICA dissection.

**Conclusions:**

COVID-19 infection can cause vessel wall vulnerability. Although patients with PD often have neck pain, ICA dissection should be considered a differential diagnosis if the patient has a recent history of COVID-19.

## Highlights

• We describe ICA dissection in a patient with Parkinson's disease after COVID-19.
• COVID-19 infection can cause vessel wall vulnerability.
• Patients with PD often have neck pain.
• However, ICA dissection should be considered if the patient has had recent COVID-19.

## Graphical abstract

**Figure:**
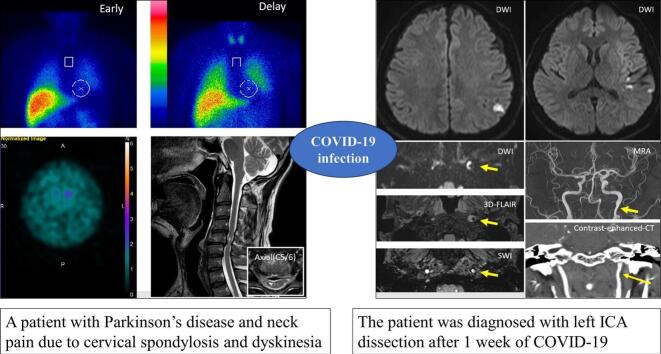

## 1 Introduction

Internal carotid artery (ICA) dissection has been reported to be a rare disease; however, the annual incidence rate has increased from 2.30 per 100,000 person-years to 8.93 per 100,000 person-years over the past 19 years because of the evolution of vascular imaging techniques [1]. However, the actual incidence may be higher in asymptomatic patients. Prior trauma is identified in only up to 40 % of cases, and most traumatic events (up to 90 %) are mild or trivial insults [2]. Stretching and compression of ICA due to sudden acceleration, deceleration, or rotational forces can increase the risk of dissection [3].

Recently, it has been proposed that severe acute respiratory syndrome coronavirus 2 (SARS-CoV-2) triggers an inflammatory response that exacerbates endothelial dysfunction leading to dissection [[4], [5], [6], [7]]. Although the cause of coronavirus disease 2019 (COVID-19) and arterial dissection is uncertain, 42 patients with arterial dissection have been reported in a recent review [4]. Here, we describe a patient with Parkinson's disease (PD) who developed levodopa-induced neck dyskinesia and cerebral infarction due to ICA dissection associated with COVID-19 infection.

## 2 Case presentation

A 51-year-old man with levodopa-induced neck dyskinesia presented to the neurology clinic with gradually worsening neck pain 1 week after a mild COVID-19 infection with symptoms including fever, coughing, and sneezing. He had experienced transient motor aphasia for approximately 2–3 h the previous day. He was clinically diagnosed with PD 6 years ago based on pre-established criteria [8]. The initial signs of PD, such as bradykinesia, rigidity, and resting tremor, were observed at 44 years of age. Levodopa improved his symptoms, but a dose increase and additional medication were needed because of the progression of PD. He exhibited neck dyskinesia during the on-state and complained of neck pain due to cervical spondylosis since the past year. The site of neck pain changed slightly to left side 1 week before presentation. He did not have any vascular risk factors such as smoking, hypertension, hyperlipidaemia, or diabetes. No family history of arterial dissection, collagen vascular disease, inherited arteriopathies, fibromuscular dysplasia, extreme vessel tortuosity, moyamoya disease, or cystic medial necrosis was noted. The PD medication included levodopa/carbidopa (150/15 mg, 5 times a day), entacapone (100 mg, twice a day), selegiline (2.5 mg twice a day), and ropinirole tape (16 mg).

**Fig. 1:**
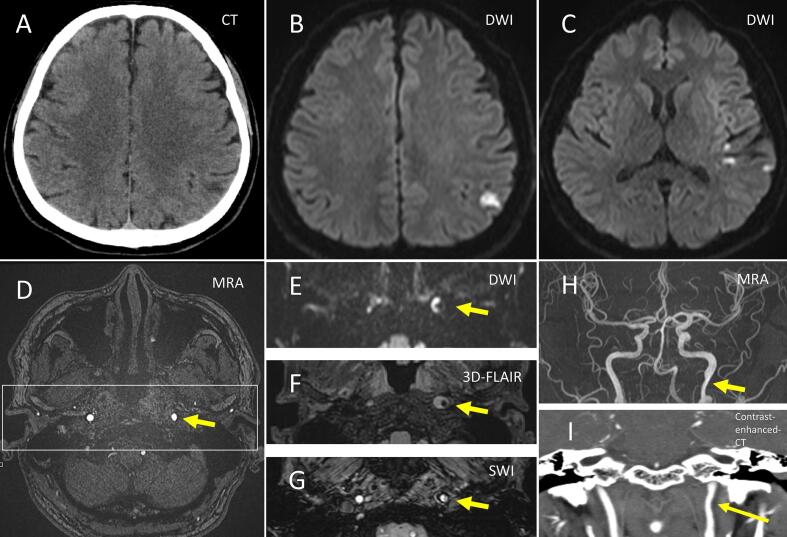
Magnetic resonance (MR) imaging (MRI) of internal carotid artery (ICA) dissection. Computed tomography (CT) reveals a low-density area in the left parietal lobe (A). This low-density area on CT is hyperintense on MRI diffusion-weighted imaging (DWI), indicating an acute infarction (B). Small hyperintensities are observed in the left hemisphere (C). The MR angiography (MRA) source image reveals stenosis of the left ICA (yellow arrow, D). In the area of stenosis, the intramural hematoma is detected as hyperintense on DWI (yellow arrow, E) and 3D-fluid attenuated inversion recovery (yellow arrow, F) and hypointense on susceptibility-weighted imaging (yellow arrow, G). These findings are compatible with those of ICA dissection. Stenosis due to dissection observed on MRA (yellow arrow, H) occurs approximately 2 cm proximal to the base of the skull (yellow arrow, I). (For interpretation of the references to colour in this figure legend, the reader is referred to the web version of this article.)

His neurological findings were normal except for parkinsonism. The Mini-Mental State Examination score was 26 (lost 4 points in series 7). Laboratory tests were normal for glucose, cholesterol, homocysteine, protein C, protein S, antithrombin III, ferritin, D-dimer, autoimmune antibodies (anti-nuclear antibodies, anti-double stranded-DNA, anti-SS-A, anti-SS-B, anti-neutrophilic cytoplasmic antibodies, anti-cardiolipin antibodies), and rheumatoid factor, and cryoglobulin. C-reactive protein was slightly elevated (0.46 mg/dL, normal <0.14 mg/dL). Liver and renal functions were normal. Brain computed tomography (CT) revealed a low-density area in the left parietal lobe (Fig. 1A). Elongated styloid processes were not observed. Additional brain magnetic resonance imaging (MRI) detected acute infarctions in the left hemisphere (Fig. 1, B and C), and an intramural hematoma was observed in an area of stenosis in the left ICA (Fig. 1, D-G). The patient was admitted with left ICA dissection. Dissection occurred approximately 2 cm proximal to the base of the skull (Fig. 1, H and I). He had no episode of migraine, and screening for other risk factors for ICA dissection, such as connective tissue abnormalities and Fabry disease, revealed unremarkable findings. Arterial fibrillation, intracardiac thrombus, and carotid artery plaque in the cervical area were not observed on electrocardiography, cardiac ultrasound, and carotid ultrasound, respectively.

**Fig. 2:**
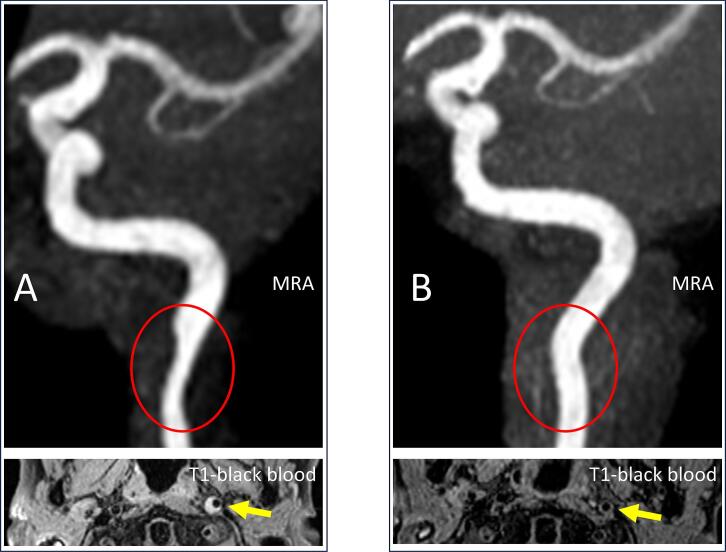
Follow-up magnetic resonance (MR) imaging (MRI). Stenosis on MR angiography and intramural hematoma on T1-weighted black blood imaging (yellow arrow) (A) shows alleviation on follow-up imaging 6 months after onset (B). (For interpretation of the references to colour in this figure legend, the reader is referred to the web version of this article.)

Antiplatelet therapy with 100 mg/day aspirin was initiated. Amantadine (100 mg/day) was initiated to treat the levodopa-induced dyskinesia. The patient's dyskinesia and neck pain improved gradually. The patient was discharged 10 days later without any sequelae and returned to work. On follow-up MRI performed 6 months later, the intramural hematoma had diminished (Fig. 2). Antiplatelet therapy was terminated in accordance with the findings in a previous study [2].

## 3 Discussion

To date, ICA dissection has not been reported in PD patients with levodopa-induced cervical dyskinesia and cervical spondylosis. Previous reports described that sudden violent neck tics or heavy exercise may be related to cervical artery dissection [3,9,10]. The mechanical stress of violent jerking motions of the head and neck has been reported as a potential risk factor for ICA dissection [3]. In the present case, the mechanical stress of cervical choreiform movement due to dyskinesia and COVID-19 may have been risk factors for the onset of ICA dissection.

According to a recent review, 42 cases of arterial dissection associated with COVID-19 were reported as of October 2021. Aortic dissection was the most common (52.3 %), followed by coronary artery dissection (23.8 %) and, less commonly, cerebral, vertebral and ICA dissections (7.1 % each) [4]. About 50 % of the patients were aged between 45 and 59 years and the period between the onset of COVID-19-like symptoms and presentation to the hospital with symptoms/signs of arterial dissection ranged from 1 day to 8 weeks [4]. The age and onset date in the present case were consistent with previous data. Common risk factors included hypertension (60 %), diabetes mellitus (15 %), dyslipidaemia (15 %), and smoking (15 %). No risk factors were identified, similar to the present case, in 47.6 % patients. Arterial dissection in patients with COVID-19 may occur secondary to an exaggerated inflammatory response that causes endothelial dysfunction. SARS-CoV-2 infects the host using the angiotensin-converting enzyme 2 receptor, which is expressed not only in the lungs and heart but also in endothelial cells [6]. Thus, endothelium throughout the body is at risk of SARS-CoV-2 [11]. The presence of SARS-CoV-2 viral particles in the capillary endothelia of a frontal lobe specimen was confirmed in an autopsy case [12]. Cervical artery dissection is rare; however, early detection and therapeutic intervention are crucial because dissecting aneurysm rupture or stroke can be life-threatening [5]. Although the relationship between ICA dissection and COVID-19 is uncertain, there are two previous case reports on ICA dissection after COVID-19 [7,13]. In the report by Asan et al., acute neck pain occurred when the patient's head was hyperextended during an oropharyngeal swab test for COVID-19 during follow-up, and MRI revealed ICA dissection [7]. Although the timing of stroke symptoms and that of worsened neck pain were close in this case, the site of dissection was atypical for traumatic ICA dissection. The dissection might have occurred because of mechanical stress at the site of endothelial damage due to SARS-CoV-2 in the present case and the previous case. The levodopa-induced dyskinesia of the neck in our case could be the mechanical stress on the cervical arteries. Coughing and sneezing also might have been the risk factors that induced dissection. Amantadine (an *N*-methyl-d-aspartate receptor antagonist), which is reported to be the most effective treatment for levodopa-induced dyskinesia, improved neck dyskinesia.

Pain is highly prevalent in patients with PD, and the neck is one of the most common sites. Cervical spinal deformity and forward head posture are associated with neck pain. A CT scan for our patient might not have been performed if he had not provided information on transient aphasia, since our patient complained of neck pain for a year, and the onset of neck pain was not acute. Supportive information for ICA dissection was that the site of his neck pain was slightly left-sided. Even in patients with PD and neck pain, cervical artery dissection may be included in the differential diagnosis if the patient has COVID-19. A major limitation of this report is that we conducted only physical/neurological examinations, blood tests, and imaging studies to rule out alternative mechanisms of ICA dissection, such as collagen vascular disease, inherited arteriopathies, fibromuscular dysplasia, extreme vessel tortuosity, or cystic medial necrosis. However, considering previous reports regarding SARS-CoV-2 and arterial dissection, further studies are needed to clarify the effect of COVID-19 infection on the vulnerability of the endothelium and arterial dissection.

## Funding

This work was supported by the grant from Okasan-Kato Foundation (funding number: 23-1-10).

## Informed consent

Informed consent was obtained from the patient for the anonymous publication of this case report.

## Data statement

Anonymized data not published within this article will be made available on request to any qualified investigator.

## Study funding

This work was supported by 10.13039/501100001691JSPS KAKENHI (grant number 21K15678).

## CRediT authorship contribution statement

**Takanobu Okubo:** Writing – original draft, Data curation. **Hidehiro Ishikawa:** Writing – review & editing, Writing – original draft, Supervision, Methodology, Investigation, Data curation, Conceptualization. **Keita Matsuura:** Writing – original draft, Supervision, Investigation. **Asako Tamura:** Writing – original draft, Supervision. **Koichi Miyashita:** Writing – original draft, Data curation. **Maki Umino:** Writing – original draft, Methodology, Data curation. **Masayuki Maeda:** Writing – original draft, Supervision, Data curation. **Akihiro Shindo:** Writing – original draft, Supervision, Project administration, Conceptualization.

## Declaration of competing interest

The authors declare that they have no conflict of interest.
